# Prostate radiation in non-metastatic castrate refractory prostate cancer provides an interesting insight into biology of prostate cancer

**DOI:** 10.1186/1748-717X-7-43

**Published:** 2012-03-22

**Authors:** Abigail C Pascoe, Santhanam Sundar

**Affiliations:** 1 Department of Oncology, Nottingham University Hospitals, City Hospital Campus, Hucknall Road, Nottingham NG5 1PB, UK; 2 University of Nottingham, University Park, Nottingham NG7 2RD, UK

**Keywords:** Prostate cancer, Prostate radiation, Castrate refractory

## Abstract

**Background:**

The natural history of non-metastatic castrate refractory prostate cancer is unknown and treatment options are limited. We present a retrospective review of 13 patients with locally advanced or high risk prostate cancer, initially treated with hormone monotherapy and then treated with prostate radiation after becoming castration refractory.

**Findings:**

Median PSA response following prostate radiation was 67.4%. Median time to biochemical progression following radiotherapy was 15 months and to detection of metastatic disease was 18.5 months. Median survival from castration resistance (to date of death or November 2011) was 60 months, with median survival from RT 42 months.

**Conclusion:**

Prostate radiation appears to be beneficial even in patients with potential micrometastatic disease, which supports the hypothesis that the primary tumour is important in the progression of prostate cancer. These results are an interesting addition to the literature on the biology of prostate cancer especially as this data is unlikely to be available in the future due to combined prostate radiation and androgen deprivation therapy now being the standard of care.

## Introduction

The combination of androgen deprivation therapy and prostate radiation (RT) is currently the standard of care for high risk and locally advanced prostate cancer patients. But prior to the publication of MRC PR07 and the SPCG-7/SFUO-3 trial results showing a survival benefit from adding radical radiotherapy to androgen deprivation therapy [1,2], primary hormone monotherapy was a treatment option for selected cases of locally advanced and high risk prostate cancer [3,4]. Adding prostate radiation for these patients when they become castration refractory (and still have radiologically localised cancer) is controversial, but there is some evidence that patients with locally advanced, castration refractory prostate cancer (CRPC) may benefit from prostate radiation [5].

The natural history of non-metastatic CRPC is unknown, and treatment options are limited. Metastatic CRPC is associated with poor prognosis; average survival being 12-18 months, which with PSA lead time may be an underestimate [6].

## Patients

We undertook a retrospective review of 13 patients from follow up clinics who received RT for localised CRPC. All 13 patients received their first hormone treatment between 1996 and 2005 under the urology team and were referred for oncology opinion after castration resistance; the majority of patients were started on 2^nd ^line or even 3^rd ^line hormones prior to referral to the oncology team and then required staging investigations, hence the delay between castration resistant state and start of radiotherapy. At the time of diagnosis, 5 patients were deemed to be unsuitable for radical treatment due to their high PSA level, 2 patients were given the choice of radiotherapy but chose hormone monotherapy, one patient was having chemotherapy for lymphoma and for the other 5 patients the reasons for choice of hormone monotherapy was unclear; inadequate documentation being one of the constraints of a retrospective review. 10 patients received LHRH agonist therapy as primary hormone treatment, 2 received diethylstilboestrol and 1 patient had surgical castration. Our institution also recruited patients to the MRC PR07 trial comparing primary hormone monotherapy vs hormones plus RT combination therapy and these patients were treated outside that trial. Prior to radical RT all patients had imaging to exclude metastatic disease, 61% of patients had CT body, 85% had bone scan and 46% had both.

Castration refractory state was defined as first increase in PSA of 2 above nadir; median (range) time to castration refractory state was 44 months (14-140). The characteristics of prostate cancer at diagnosis were: Gleason score: 5 = 1; 6 = 4; 7 = 5; 9 = 1; no information was available for 2 patients; T1 = 1; T2 = 7; T3 = 3, T4 = 1; no T stage was available for 1 patient. Median (range) PSA at diagnosis was 51.85 (19-593). Patients received prostate radiation with a median dose of 64Gy (range 60-70) in 32# (30-38). Median (range) age at time of diagnosis was 63 (54-77) and at start of RT was 73 (64-81) and median (range) PSA prior to RT was 16(2.1-163). All patients continued with LHRH agonists during and after radiotherapy; 46% of patients had concurrent maximal androgen blockade with bicalutamide during and after radiotherapy, 31% had concurrent prednisolone and 1 patient had ketoconazole and hydrocrotisone, while 1 patient had LHRH agonist alone. Following biochemical relapse, patients were treated with further hormone manipulation; 6 patients received chemotherapy with docetaxel following diagnosis of metastatic disease.

## Results

Median PSA response was 67.4% (range 0-99.5%). Biochemical progression following RT was defined as first increase in PSA of 2 above post-RT nadir; median time after RT to biochemical progression was 15 (range 0-49) months, and to detection of metastatic disease was 18.5 (range 4-67) months. Median survival from castration resistance (to date of death or November 2011) was 60 (range 49-106) months, with median survival from RT 42 (range 23-77) months, only 4 of the 13 patients have died so further follow up may show improved survival data. There was no clinical local failure.

SPSS18 statistics software was used to calculate Spearmans rank correlation and showed that PSA pre-radiotherapy was negatively correlated with times to progression and development of metastatic disease (time to progression r = -0.669, p = 0.017; time to metastases, r = -0.667, p = 0.025). Percentage PSA response was positively correlated with both time to progression and time to metastatic disease (time to progression r = 0.813, p = 0.001; time to metastases r = 0.717, p = 0.013). PSA is therefore likely to be a useful marker of outcome in CRPC treated with RT; although neither of the correlations remained significant with overall survival, this may be due to the fact 9 patients in the series are still alive.

## Conclusion

With the caveats associated with a retrospective study, we conclude that prostate radiation has a clinical benefit for this sub group of patients. The benefits of prostate radiation in patients with localised CRPC are twofold: for local control of cancer, to prevent distressing local symptoms and to delay eventual overall disease progression. Clinically in this series there were no events of local failure although post-radiation prostate biopsy was not done to confirm this; radiotherapy was well tolerated with no episodes of grade 3 or 4 toxicity. In terms of distant failure, we hypothesise that prostate 'resident' stem cells may influence the natural history of prostate cancer even in the presence of micrometastases. A large retrospective study by Oefelein et al [6] reported that the median survival after the development of hormone-refractory disease was 40 months in the subgroup of patients with evidence of skeletal metastasis at diagnosis and 68 months in the subgroup without skeletal metastasis. The survival of patients in our series is comparable to that reported by Oefelein and thereby rules out significant selection bias inherent with a retrospective study; of note, patients in that study were taken to have CRPC from the time of PSA rise above 0.3, whereas our study used the RTOG-ASTRO definition of CRPC (PSA of 2 + nadir) [7], suggesting a lead time on their survival data when compared to our series.

These results show that prostate radiation may be beneficial even in the presence of potential micro metastases in these high risk patients, suggesting that the primary tumour may be a factor in promoting progression. This case series therefore provides support for the hypothesis that potential prostate cancer stem cells within the prostate play a part in the evolution of the disease in the presence of micrometastases [8]; thus also providing indirect support to the proposed EORTC trial exploring the role of prostate radiotherapy in patients with newly diagnosed metastatic prostate cancer (Proposed EORTC trial: SELECTION: Systemic and local therapy for newly diagnosed M1 prostate cancer: investigating the role of up-front cabazitaxel and irradiation of the prostate).

This data set is a unique and interesting addition to the literature on the biology of prostate cancer, as combined prostate irradiation and androgen deprivation therapy is now standard care, and therefore it is very unlikely that this information on the natural history of prostate cancer will be available in the future.

## Competing interests

S Sundar has attended Sanofi-Aventis advisory board meetings and has received honoraria, conference sponsorship, research funding from Sanofi-Aventis. A Pascoe has received conference sponsorship from Sanofi-Aventis.

## Authors' contributions

SS conceived the idea for the retrospective review and was involved in critically revising drafts of manuscript. AP collated, analysed and interpreted data; drafted manuscript and revised drafts of manuscripts. Both authors read and approved final version of manuscript.
